# A major interspecies difference in the ionic selectivity of megakaryocyte Ca^2+^-activated channels sensitive to the TMEM16F inhibitor CaCCinh-A01

**DOI:** 10.1080/09537104.2019.1595560

**Published:** 2019-04-22

**Authors:** Kirk A. Taylor, Martyn P. Mahaut-Smith

**Affiliations:** 1 Department of Molecular and Cell Biology, University of Leicester, Leicester, UK; 2 National Heart and Lung Institute, Cardio-respiratory Section, Imperial College London, London, UK

**Keywords:** Calcium, megakaryocyte, phospholipid scrambling, platelet, Scott syndrome, TMEM16F

## Abstract

TMEM16F is a surface membrane protein critical for platelet procoagulant activity, which exhibits both phospholipid scramblase and ion channel activities following sustained elevation of cytosolic Ca^2+^. The extent to which the ionic permeability of TMEM16F is important for platelet scramblase responses remains controversial. To date, only one study has reported the electrophysiological properties of TMEM16F in cells of platelet/megakaryocyte lineage, which observed cation-selectivity within excised patch recordings from murine marrow-derived megakaryocytes. This contrasts with reports using whole-cell recordings that describe this channel as displaying either selectivity for anions or being relatively non-selective amongst the major physiological monovalent ions.

We have studied TMEM16F expression and channel activity in primary rat and mouse megakaryocytes and the human erythroleukemic (HEL) cell line that exhibits megakaryocytic surface markers. Immunocytochemical analysis was consistent with surface TMEM16F expression in cells from all three species. Whole-cell recordings in the absence of K^+^-selective currents revealed an outwardly rectifying conductance activated by a high intracellular Ca^2+^ concentration in all three species. These currents appeared after 5–6 minutes and were blocked by CaCC_inh_-A01, properties typical of TMEM16F. Ion substitution experiments showed that the underlying conductance was predominantly Cl^–^-permeable in rat megakaryocytes and HEL cells, yet non-selective between monovalent anions and cations in mouse megakaryocytes. In conclusion, the present study further highlights the difference in ionic selectivity of TMEM16F in platelet lineage cells of the mouse compared to other mammalian species. This provides additional support for the ionic “leak” hypothesis that the scramblase activity of TMEM16F does not rely upon its ability to conduct ions of a specific type.

## Introduction

Procoagulant activity resulting from exposure of anionic membrane phospholipids is critical for thrombosis and haemostasis. Although the underlying mechanisms of this lipid redistribution process are incompletely understood, a Ca^2+^-activated phospholipid scramblase is known to be important [1–3]. TMEM16F is a ubiquitously expressed Ca^2+^-dependent ion channel and phospholipid scramblase [2,4]. Missense mutations of the TMEM16F gene occur in patients with Scott syndrome, a rare bleeding diathesis characterised by defective Ca^2+^-dependent phospholipid scrambling [5–7]. Furthermore, this disease is phenocopied in TMEM16F^−/−^ mice [3,8].

The nature of the ionic permeability of TMEM16F channels is controversial. Studies of native cells and expression systems conclude that human and murine TMEM16F channels are predominantly anion-permeable [4,9–11]. However, multiple reports demonstrate that heterologously expressed TMEM16F displays significant permeability to monovalent cations (P_Na_/P_Cl_: 0.3 [12] 0.5 [13] and 1.38 [14]). Such studies have principally relied upon the whole-cell patch clamp configuration. Contrastingly, using excised inside-out membrane patch recordings, one group has reported that murine TMEM16F forms non-selective cation channels with a greater permeability to Ca^2+^ than monovalent cations both endogenously in the native megakaryocyte (MK) and in expression systems [8,15]. This raises the question of whether the properties of TMEM16F are influenced by patch excision or by the environment of a specific cell type. MKs are responsible for generating all proteins within their anuclear product and exhibit functional platelet responses [16,17], thus are frequently used as a surrogate for electrophysiological studies of the tiny, fragile platelet.

We have employed whole-cell patch clamp recordings to investigate the biophysical and pharmacological properties of Ca^2+^-activated TMEM16F channels in freshly isolated primary rat and mouse MKs and the human erythroleukemia (HEL) cell line. HEL cells express a number of megakaryocytic glycoproteins [18], have been used for the study of native MK ion channels and express TMEM16F transcripts [19].

## Methods

*Ethics:* Ethical Approval for this study was granted by the University of Leicester College of Life Sciences Research Ethics Committee for Human Biology (non-NHS).

*Primary MK isolation:* MKs were prepared as previously described [20,21] from adult Wistar rats and C57bl/6 mice following euthanasia in accordance with the UK Animals (Scientific Procedures) Act 1986.

*Cell culture:* HELs (ATCC, Middlesex, UK) were cultured in RPMI 1640 (Invitrogen, Paisley, UK) supplemented with foetal calf serum (10%; Invitrogen) and penicillin/streptomycin (250U/mL; Invitrogen).

*Immunocytochemistry:* Cell suspensions were prepared as described previously [22]. Samples were incubated with anti-TMEM16F primary (2µg/ml; Santa Cruz, California, USA) and alexafluor647 (AF647)-conjugated secondary antibodies (1:1000; Invitrogen). Fluorescence was assessed with an Olympus FV1000 confocal microscope (635nm excitation, 650-750nm emission; Olympus, UK).

*Electrophysiology:* Whole-cell patch clamp recordings were conducted as described previously with ≥70% series resistance compensation and *a* *priori* liquid junction potential correction [23]. Bath solutions contained 150mM NaCl, 1mM CaCl_2_, 1mM MgCl_2_, 10mM glucose, 10mM HEPES (pH 7.35; NaOH). Where indicated, [Cl^−^]_o_ and/or [Na^+^]_o_ were reduced by equimolar substitution with gluconate^−^ or NMDG^+^, respectively. Internal solutions contained 150mM NaCl, 1mM MgCl_2_, 10mM glucose, 10mM HEPES, 50µM Na_2_-GTP, 1mM EGTA (pH 7.2; NaOH). [Ca^2+^]_i_ was set at ≈5nM (1mM EGTA, no added Ca^2+^) or 100µM (by addition of CaCl_2_), calculated using Maxchelator (http://web.stanford.edu/~cpatton/webmaxcS.htm). The effect of CaCC_inh_-A01 (A01; Merck, Watford, UK) was compared with vehicle (DMSO) control. Statistical analysis was by two-way ANOVA (Prism7, GraphPad Software Inc., CA, USA).

## Results

**Figure 1.**
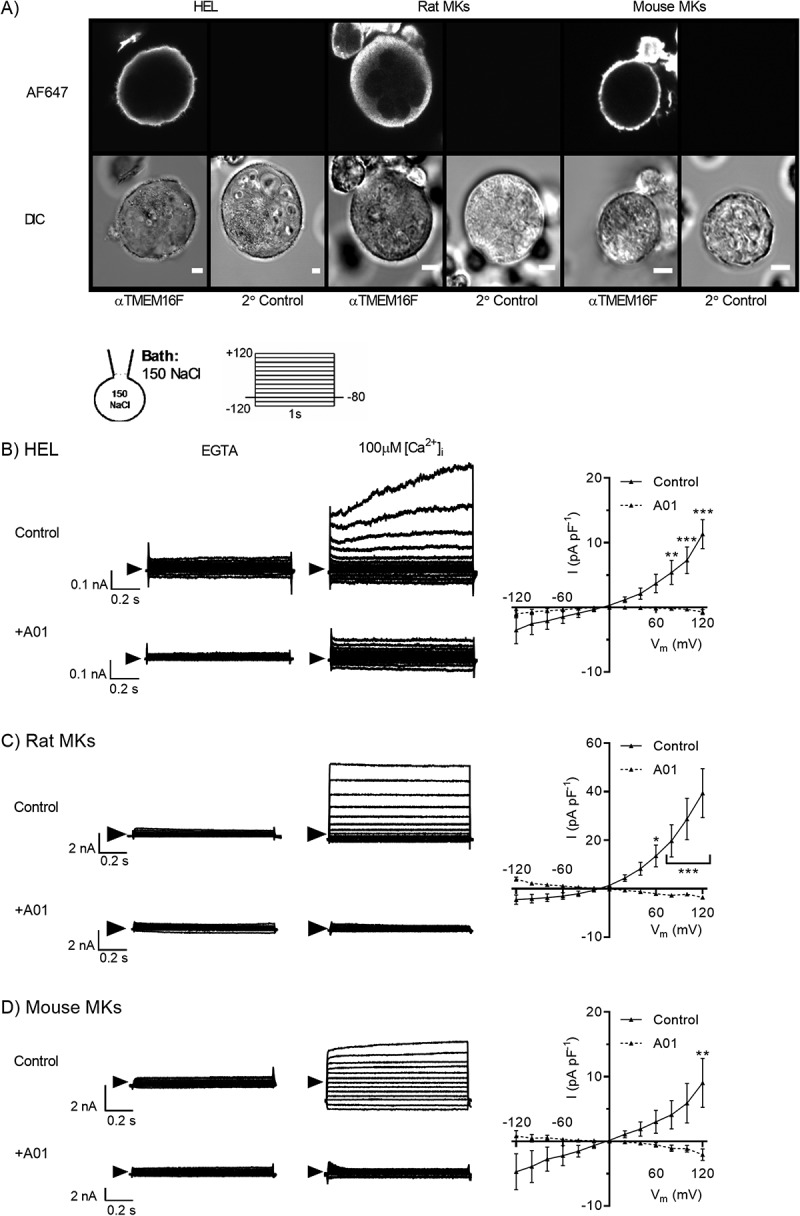
**Detection of Ca^2+^-activated and A01-sensitive TMEM16F-like currents in HEL cells and rat and mouse MKs**. A) TMEM16F expression by permeabilised HEL cells (left), primary rat (centre) and mouse (right) MKs assessed by immunocytochemistry with a primary antibody raised against an intracellular epitope of TMEM16F. Strong fluorescence was observed at the periphery of cells treated with primary (αTMEM16F) and secondary (AF647) antibodies, whilst no fluorescence was detected in secondary antibody only controls. B-D) whole-cell patch clamp recordings of Ca^2+^-activated currents from HEL cells (B), rat (C) and mouse (D) MKs. Intracellular and bath solutions contained 150mM NaCl and were K^+^-free. [Ca^2+^]_i_ was set at either ≈5nM (1mM EGTA) or 100µM as indicated. After 10 minutes in the whole-cell mode, currents were recorded in response to voltage steps of 1s duration (−120 to +120mV, 20mV increments) in the presence of vehicle control (0.04% DMSO) or the TMEM16F antagonist CaCCinh-A01 (A01; 20µM). Representative traces are shown for control or A01-treated MKs in the presence of intracellular EGTA or 100µM [Ca^2+^]_i_. Summary current density-voltage relationship data are shown in the right hand panels for EGTA-subtracted currents under control (solid line) or A01-treated (dashed line) conditions. For immunocytochemistry experiments, scale bars represent 10µm. Data are representative of a minimum of three independent experiments for each condition. *, ** and *** denote *p < 0.05, p < 0.01* and *p < 0.001*, respectively.

TMEM16F expression in HELs and rat and mouse MKs was assessed by immunocytochemistry with an antibody previously used in mouse dendritic cells [9]. Fluorescence was detected in primary MKs from both species and HELs, with a pattern indicating strong surface expression and no signal from secondary antibody-only controls (Figure 1A).

Previous electrophysiological studies demonstrate that primary MKs and HELs display robust K^+^ currents activated by depolarisation and/or intracellular Ca^2+^ [23–26]. Within whole-cell recordings from other cell types, TMEM16F channels typically activate in response to sustained (5–6 min) elevation of high [Ca^2+^]_i_ (EC_50_ of ≈100μM) [12,14]. Therefore, in our study, K^+^-free bath and pipette salines were used to abolish K^+^ currents prior to the predicted activation of TMEM16F. Voltage ramps from −120 to +120mV were applied at regular intervals and [Ca^2+^]_i_ set at either ≈5nM or 100µM. At 100µM [Ca^2+^]_i_, outwardly rectifying currents developed at similar timepoints in cells from all species: 439 ± 25s (n = 5) in HELs, 418 ± 18s (n = 5) in rat MKs and 368 ± 20s in mouse MKs (n = 4). This current was not observed at ≈5nM [Ca^2+^]_i_ and its activation time course is consistent with reports of TMEM16F [12,14]. After 10 minutes, 1s voltage steps were applied across the range −120 to +120mV, as previously used for further characterisation of TMEM16F [8,12]. In the rat MK, 100µM [Ca^2+^]_i_ induced large outwardly rectifying currents that reversed (E_rev_) at −3.3 ± 0.5mV (Figure 1C) and were blocked by the TMEM16F antagonist A01 [8,9,27,28] (39.4 ± 3.8 vs −3.6 ± 0.7pA/pF at +120mV; P < 0.001, n = 5, Figure 1C). The conductance in HELs and mouse MKs possessed similar properties, although the current amplitudes and extent of outward rectification were smaller in these species compared to the rat. The current amplitude at +120mV compared to −120mV was 18.8 ± 3.1 (n = 5), 43.9 ± 10.3 (n = 5) and 13.7 ± 4.7 (n = 4) in HELs, rat and mouse MKs, respectively. In HELs the Ca^2+^-activated currents reversed at −1.7 ± 0.3 mV and were reduced from 14.1 ± 2.3 to 2.0 ± 0.4 pA/pF at +120 mV by A01 (Figure 1B). For mouse MKs, Ca^2+^-induced currents reversed close to 0 mV and were reduced from 9.0 ± 3.8 to −1.0 ± 0.8 pA/pF at 120mV by A01; P < 0.01, n = 4 [control] and n = 3, Figure 1D).

**Figure 2.**
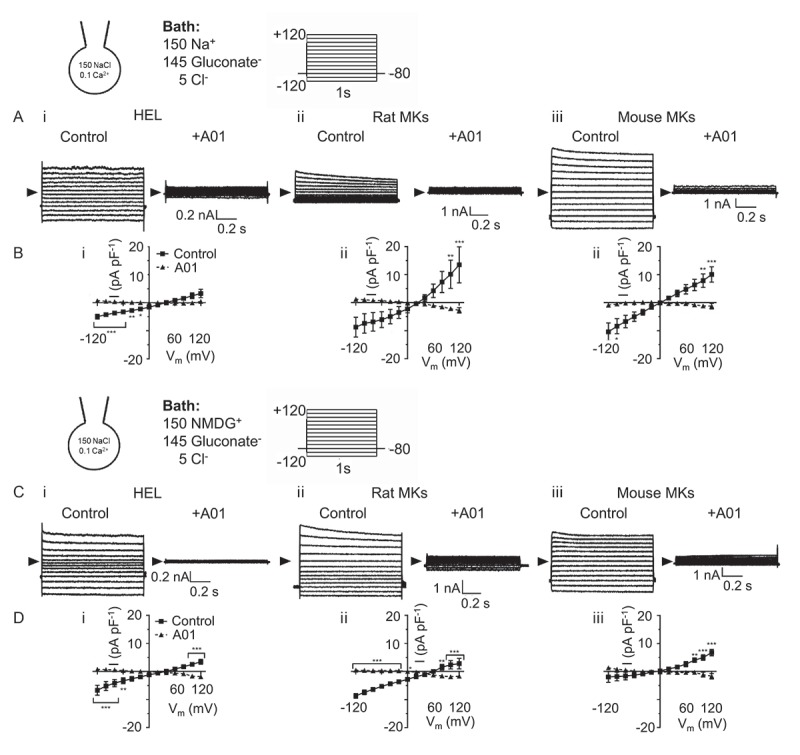
**Comparison of ionic selectivity between human, rat and mouse megakaryocytic Ca^2+^-activated, A01 sensitive TMEM16F-like channels**. Whole-cell patch clamp recordings of Ca^2+^-activated currents from HEL cells (left), rat (centre) and mouse (right) MKs under conditions designed to isolate TMEM16F channel activity. Intracellular solutions were K^+^-free (150mM NaCl), and membrane currents were assessed after 10 mins in the whole cell configuration with [Ca^2+^]_i_ set at either ≈5nM (1mM EGTA, not shown) or 100µM. Currents were recorded in response to voltage steps of 1s duration (−120 to +120mV, 20mV increments) in the presence of vehicle control (0.04% DMSO) or the TMEM16F antagonist CaCCinh-A01 (A01; 20µM). For experiments in panels A-B extracellular Cl^−^ was substituted by equimolar gluconate^−^ and in panels C-D, extracellular Na^+^ and Cl^−^ were substituted by NMDG^+^ and gluconate, respectively. B,D) Summary current density-voltage relationship data for Ca^2+^-activated currents under vehicle control (solid line) or A01-treated (dashed line) conditions. Data are representative of a minimum of three independent experiments for each condition. *, ** and *** denote *p < 0.05, p < 0.01* and *p < 0.001*, respectively.

Under the ionic conditions of Figure 1, the equilibrium potential for both Na^+^ and Cl^−^ is 0mV and thus Ca^2+^-activated currents may reflect movement of cations and/or anions. Following reduction of external chloride (gluconate substitution), 100µM [Ca^2+^]_i_ evoked large A01-sensitive currents in each species (Figure 2A). Strikingly, E_rev_ shifted in HELs (+37.2 ± 2.1mV) and rat MKs (+33.5 ± 2.7mV), whereas mouse MK currents continued to reverse close to 0mV (Figure 2B). Under low [Cl^−^]_o_ conditions E_Na_ = 0mV and E_Cl_ = +85.7mV, indicating that the underlying conductance displays greater permeability to Cl^−^ compared to other ions in HELs and rat MK, but not in the mouse MK. A lower, yet significant, permeability to cations or large anions may explain why the shift of E_rev_ was less than that expected for a perfectly Cl^–^-selective conductance, as reported previously for Cl^−^ channels in human platelets [29,30].

In low Cl^−^ external saline, substitution of [Na^+^]_o_ with the large cation NMDG^+^ failed to alter E_rev_ in mouse MKs but shifted this value to a slightly more positive potential in rat MKs (+41.9 ± 3.4mV) and HELs (+40.4 ± 3.9mV; Figure 2C,D). These data further suggest a major difference in the ionic selectivity of the TMEM16F-like conductance in megakaryocytic cells from mouse compared to rat or human. They also indicate that the mouse channel is highly non-selective amongst the major monovalent ions used in this study.

## Discussion

The ionic selectivity of Ca^2+^-activated TMEM16F channels has been variably reported as anionic [4,9–12], cationic [8,15] and non-selective between monovalent anions and cations [13,14]. Here, we compared the ionic selectivity of the Ca^2+^-activated conductances of primary megakaryocytes from rat and mouse, and a human megakaryocyte cell line, under conditions previously used to characterise TMEM16F. We observed that endogenous TMEM16F-like conductances are relatively selective for Cl^−^ in rat MKs and a human megakaryocytic cell line but are non-selective in mouse MKs (Figure 2). Although cation-selective TMEM16F channels have been reported in the mouse MK [8] and following heterologous expression of the murine clone in oocytes and HEK cells [8,15], a study of mouse dendritic cells concluded that this conductance is more permeable to Cl^−^ than cations [9]. Plausible explanations for this difference include cell-specific expression of alternative splice variants [13,31] or an effect of the cell environment on channel properties. A further possibility is that the channel may behave differently in excised inside-out patches, which have been exclusively used in studies that conclude TMEM16F is cation-selective [8,15], compared to whole-cell recordings used for most other studies. Interestingly, in excised patch recordings, TMEM16F was also observed to show greater permeability to Ca^2+^ than monovalent cations, which may play a role in scramblase activation or inactivation [8,15]. We were unable to test the relative Ca^2+^ permeability of the channel due to poor viability of whole-cell recordings in low or high [Ca^2+^]_o_ solutions. Nevertheless, it seems unlikely that Ca^2+^ entry through TMEM16F is crucial for its own activation since this requires sustained high [Ca^2+^]_i_ levels. It has also been proposed that Cl^–^-selective TMEM16F channels cause membrane hyperpolarisation, thereby enhancing Ca^2+^ influx through an increased driving force [28]. However, activated platelets have a negative membrane potential (−45mV to −80mV) set by voltage-gated or Ca^2+^-activated K^+^ channels [23,30,32] which drives Ca^2+^ influx primarily via Orai1, P2X1 and TRPC6 [23,30,33]. Since the platelet Cl^−^ equilibrium potential has been estimated to sit at ≈-35mV [30], activation of either a Cl^–^-selective or non-selective channel (E_rev_≈0mV) would depolarise rather than hyperpolarise the cell. This argues against a role for TMEM16F in promoting Ca^2+^ entry via regulation of the membrane potential.

Although we demonstrate an interspecies difference of ion selectivity for MK TMEM16F-like channels, further work is required to understand the functional significance of the resultant ionic movements for lipid scrambling and/or other functional events in this cell type. Furthermore, the possible relevance of expression of other members of this scramblase/channel family should also be examined with knock-down studies. It is worth noting however that a transcriptomic analysis of purified platelets detected <2% expression of other TMEM16 family members compared to TMEM16F [19]. Furthermore, the same study detected only TMEM16K in addition to TMEM16F in HEL cells, and TMEM16K is inhibited at the high intracellular Ca^2+^ concentrations used in the present work [4]. A study of HEK293 cells reported that TMEM16F-dependent currents and phosphatidylserine exposure occur contemporaneously, and that preventing net ionic movement by clamping the membrane voltage to zero does not affect phosphatidylserine scrambling [14]. Furthermore, despite the species difference in ionic selectivity between mouse and human MK TMEM16F, it is worth noting the similarities between the altered scramblase activity of TMEM16F^−/−^ mice [3,8] and Scott Syndrome patients [1]. This suggests that the ability to conduct specific ionic species, or an influence on membrane potential, are unlikely to be crucial determinants of TMEM16F-mediated lipid scrambling and thus platelet procoagulant activity. Future studies should therefore directly assess the relationship between platelet and/or megakaryocyte lipid scrambling and TMEM16F ionic selectivity. Overall, the present work provides further support for the “leak” hypothesis of ionic movement through TMEM16F, in which ions move because of phospholipid translocation rather than being critical for lipid redistribution [14].
